# Using viromes to predict novel immune proteins in non-model organisms

**DOI:** 10.1098/rspb.2016.1200

**Published:** 2016-08-31

**Authors:** Steven D. Quistad, Yan Wei Lim, Genivaldo Gueiros Z. Silva, Craig E. Nelson, Andreas F. Haas, Linda Wegley Kelly, Robert A. Edwards, Forest L. Rohwer

**Affiliations:** 1 Department of Biology, San Diego State University, 5500 Campanile Drive, San Diego 92182, USA; 2 Computational Science Research Center, San Diego State University, 5500 Campanile Drive, San Diego 92182, USA; 3 Department of Computer Science, San Diego State University, 5500 Campanile Drive, San Diego 92182, USA; 4 Center for Microbial Oceanography: Research and Education, Department of Oceanography and Sea Grant College Program, School of Ocean and Earth Science and Technology, University of Hawai‘i at Mānoa, HI 96822, USA

**Keywords:** viruses, evolution, immunity, coral, cnidarians, comparative genomics

## Abstract

Immunity is mostly studied in a few model organisms, leaving the majority of immune systems on the planet unexplored. To characterize the immune systems of non-model organisms alternative approaches are required. Viruses manipulate host cell biology through the expression of proteins that modulate the immune response. We hypothesized that metagenomic sequencing of viral communities would be useful to identify both known and unknown host immune proteins. To test this hypothesis, a mock human virome was generated and compared to the human proteome using tBLASTn, resulting in 36 proteins known to be involved in immunity. This same pipeline was then applied to reef-building coral, a non-model organism that currently lacks traditional molecular tools like transgenic animals, gene-editing capabilities, and *in vitro* cell cultures. Viromes isolated from corals and compared with the predicted coral proteome resulted in 2503 coral proteins, including many proteins involved with pathogen sensing and apoptosis. There were also 159 coral proteins predicted to be involved with coral immunity but currently lacking any functional annotation. The pipeline described here provides a novel method to rapidly predict host immune components that can be applied to virtually any system with the potential to discover novel immune proteins.

## 1. Introduction

Comparative immunology uses a variety of invertebrate and vertebrate species to better understand the evolution and origin of immunity [1]. These model organisms have provided fundamental insights into evolutionarily conserved immune mechanisms. For example, the initial concept of self versus non-self recognition was formed by experimentation with starfish, and studies in chickens led to the identification of separate B and T cell lineages [2,3]. While these discoveries have been invaluable in understanding immune mechanisms, the majority of comparative immunology is based on data from only 3 of the 30 extant animal phyla [2]. A broader representation of phyla will help to fully appreciate the complexity of immunity.

Currently, the establishment of molecular tools is infeasible in terms of time and money for most of the metazoan tree of life. For example, continuous cell lines have been a long-term goal of coral reef biologists for decades. While there have been reports of primary cell cultures, standardized cell lines have yet to be established and genetic manipulation of corals remains elusive [4–7]. To investigate the immune systems of corals, as well as the remaining twenty-seven other animal phyla, a broad and rapid approach is required [2]. Here, we propose a comparative genomic approach in which host viral communities are used to predict host immune proteins.

Viruses are dependent on a host cell to complete their life cycle. Therefore, they must replicate and package their genomes intracellularly while avoiding the host immune response. To establish and maintain control of host cells, viruses mimic host proteins [8–11]. For example, herpes viruses encode proteins that possess sequence similarity to human cytokines and can modulate cytokine signalling in host cells during infection [12]. If a herpesvirus-encoded cytokine was compared with the human proteome using BLAST, then the human-encoded cytokine would be identified without any *a priori* knowledge of the human immune system. Expanding this observation, we hypothesized that an *in silico* comparison of all viral gene products to the predicted host proteome would identify both known and unknown immune-associated proteins.

As a proof of principle, *in silico* constructed viromes were used to successfully identify known human immune proteins. This method was then applied to reef-building corals to identify proteins potentially involved in coral immunity. Most of these proteins were homologues to human proteins, but a subset of 159 coral proteins were predicted to be involved in novel coral immune functions. This method generates putative host immune proteins to be further tested with the goal of discovering new types of immunology.

## 2. Results

### (a) Known viruses versus the well-characterized human immune system

**Figure 1.**
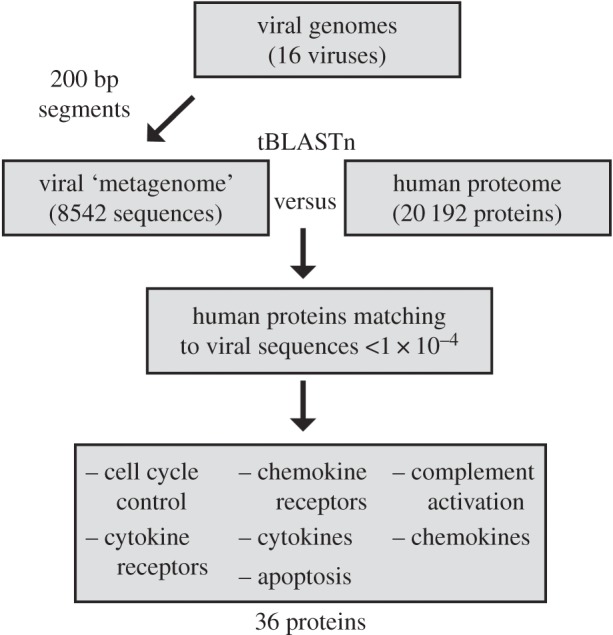
Bioinformatic pipeline used to identify viral gene segments with sequence similarity to host proteins: characterized viruses versus characterized immune system (humans). Virus genomes were segmented into 200 base pair sequences to create a mock viral metagenome and compared with the human proteome using tBLASTn analysis with an *e*-value cutoff of less than 1 × 10^−4^. Viruses included human herpesviruses 1–3, 5, 6A, 6B, 7–8, human adenoviruses A-E, human circovirus, and human papillomaviruses 1, 2. See electronic supplementary material, table S1*a*, for description of viruses used in the analysis, electronic supplementary material, table S1*b*, for summary of human proteins identified, and electronic supplementary material, file S1 for full tBLASTn results.

**Figure 2.**
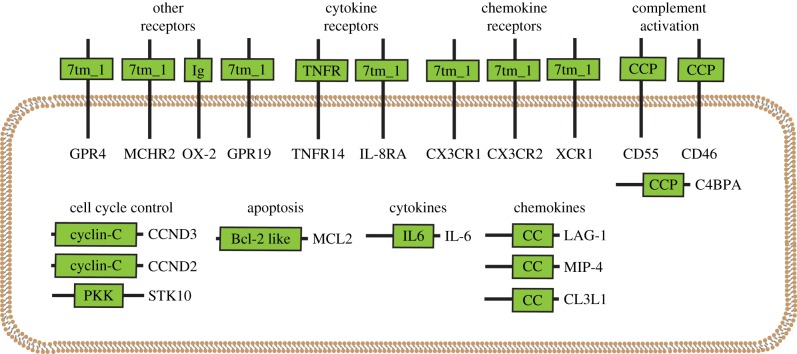
Proteins identified using tBLASTn analysis of mock viral metagenome versus human proteome grouped by function. Green boxes indicate domains within a specific protein with gene names indicated. 7tm_1, 7 transmembrane receptor (Rhodopsin family); Ig, immunoglobulin; TNFR, tumour necrosis factor receptor; CCP, complement control protein; PKK, polo kinase kinase; Bcl-2 like, B-cell lymphoma-2 family; IL6, interleukin 6; CC, chemokine. See electronic supplementary material, table S1*b* for summary of human proteins identified, and electronic supplementary material, file S1 for full tBLASTn results.

As a test, the proposed method was applied to viruses known to manipulate the human immune system (figure 1). A mock virome was created by fragmenting 16 fully sequenced viral genomes into 200 base pair segments. The resulting *in silico* virome consisted of 8542 DNA segments (figure 1; electronic supplementary material, table S1*a*). This mock human virome was then compared with the human proteome using tBLASTn, with an *e*-value of 1 × 10^−4^ as a cutoff for significance yielding 36 human proteins matching viral DNA segments (electronic supplementary material, file S1). The function of these proteins includes cell cycle control, cytokines, cytokine receptors, chemokines, chemokine receptors, apoptosis and complement activation (figure 2; electronic supplementary material, table S1*b*).

### (b) Coral derived viromes versus predicted coral proteomes

**Figure 3.**
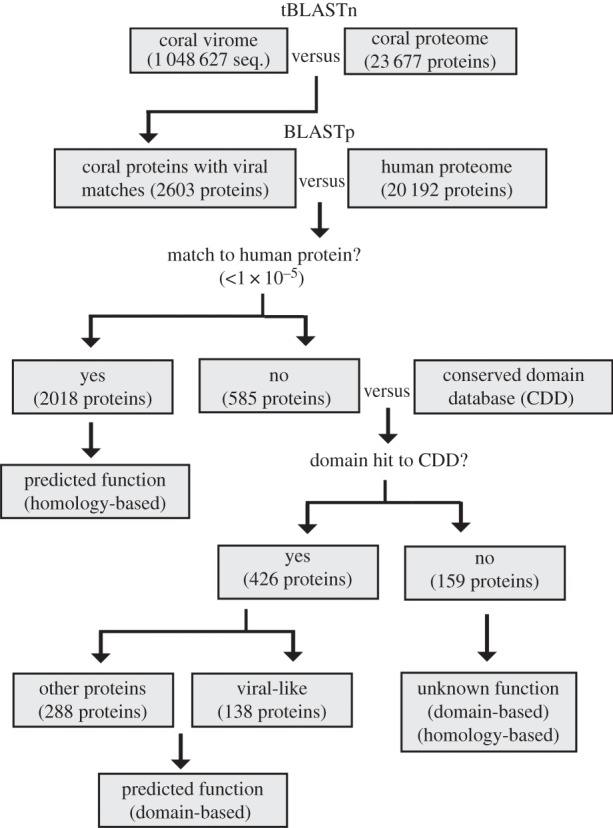
Bioinformatic pipeline used to identify viral gene segments with sequence similarity to host proteins: uncharacterized viruses versus uncharacterized immune system (e.g. corals). Coral viromes extracted from four species of coral were compared with the predicted *Acropora digitifera* proteome through tBLASTn analysis resulting in 2503 protein hits. Coral proteins were classified as ‘hits’ if five or more unique viral sequences matched with *e*-value less than 1 × 10^−4^ (see electronic supplementary material, table S3 for a summary of viral hits to coral proteins and electronic supplementary material, file S2 for full tBLASTn results). To determine whether the function of the 2503 coral proteins could be predicted based on sequence similarity to human proteins they were compared with the human proteome using BLASTp with an *e*-value cutoff of 1 × 10^−5^ resulting in 2018 coral proteins with positive matches to human proteins (see electronic supplementary material, file S3 for full BLASTp results). To determine whether any of the remaining 585 proteins function could be predicted based on the presence of conserved domains they were analysed using the conserved domain database (CDD) resulting in 426 coral proteins with positive matches to a known domain (see electronic supplementary material, file S4 for full results of CDD analysis). Within this group of proteins 138 were considered ‘viral-like’ based on the presence of domains associated with viral proteins. In total 159 coral proteins possessed no similarity to either database and therefore represent predicted immune proteins with unknown function.

**Figure 4.**
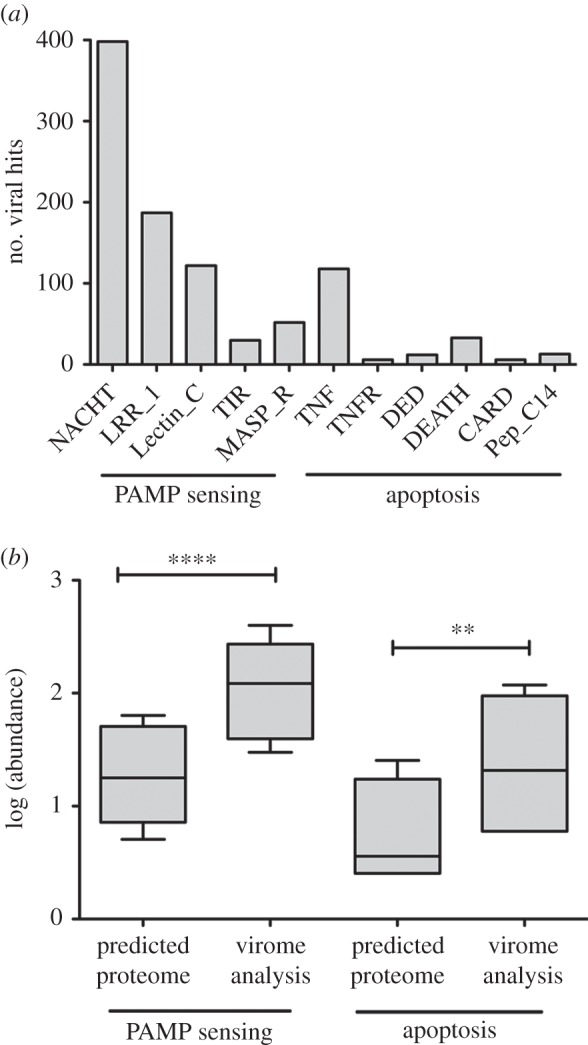
Frequency of apoptotic and pathogen-associated molecular pattern (PAMP) domains within the predicted coral proteome (23 677 proteins) and the group of coral proteins identified through virome analysis (2503 proteins). (*a*) Total number of viral hits to coral proteins containing domains related to PAMP sensing and apoptosis. PAMP domains include NAIP (neuronal apoptosis inhibitor protein), C2TA (MHC class 2 transcription activator), HET-E (incompatibility locus protein from *Podospora anserina*) and TP1 (telomerase-associated protein) (NACHT), leucine-rich repeat (LRR_1), C-type lectin (Lectin_C), toll-interaction region (TIR), and mannose binding lectin associated serine protease (MASP_R). Apoptotic domains include tumour necrosis factor (TNF), tumour necrosis factor receptor (TNFR), death effector domain (DED), caspase activation and recruitment domain (CARD) and peptidase C14 (Pep_C14). (*b*) Abundance of PAMP sensing and apoptosis-associated domains within the predicted coral proteome and the group of proteins identified through virome analysis. Paired two-tailed *t*-test *p*-value < 0.0001 (PAMP sensing) and 0.001 (apoptosis) (see electronic supplementary material, table S4*a* for summary table, table S4*b* for statistical analysis and table S5 for domain descriptions).

Fourteen viromes were generated from four species of coral; *Porites rus* (four colonies), *Acropora sp*. (three colonies), *A. yongeii* (three colonies) and *Pocillopora verrucosa* (four colonies) [13]. Sequence data quality control was performed using PRINSEQ and DeconSeq, resulting in 1 048 627 good-quality sequences with an average size of 315 base pairs (electronic supplementary material, table S2) [14,15]. For this analysis, the viromes were combined *in silico* into one coral virome and compared with the predicted proteome of *A. digitifera* using tBLASTn. An *e*-value of less than 1 × 10^−4^ was used as the cutoff for significance [16] (see electronic supplementary material, file S2, for full tBLASTn results). This resulted in a total of 60 191 virome-derived DNA sequences that significantly matching 5 863 predicted coral protein sequences. To reduce the complexity of this dataset, only coral proteins that matched to at least five of the virome fragments were considered for further analyses (2 503 proteins total; figure 3; electronic supplementary material, table S3) [17]. The potential functions of this group of proteins was predicted by comparing to the human proteome using BLASTp (electronic supplementary material, file S3), as well as a functional domain analysis using the conserved domain database (CDD) (electronic supplementary material, file S4) [18]. This showed that 83 proteins related to PAMP sensing and apoptosis (figure 4; electronic supplementary material, figure S1 and table S4*a*) [18]. Electronic supplementary material, table S5, provides descriptions of protein domains classified as PAMP sensing [19–23] and apoptosis [24–30]. Compared with the remainder of the coral proteome, this group of host proteins with homology to viral proteins was significantly enriched for PAMP sensing and apoptosis-associated domains (figure 4*b*; electronic supplementary material, table S4*b*; paired two-tailed *t*-test *p*-values ≤ 0.0001 and 0.001, respectively).

### (c) Bioinformatic prediction of novel immune proteins

**Figure 5.**
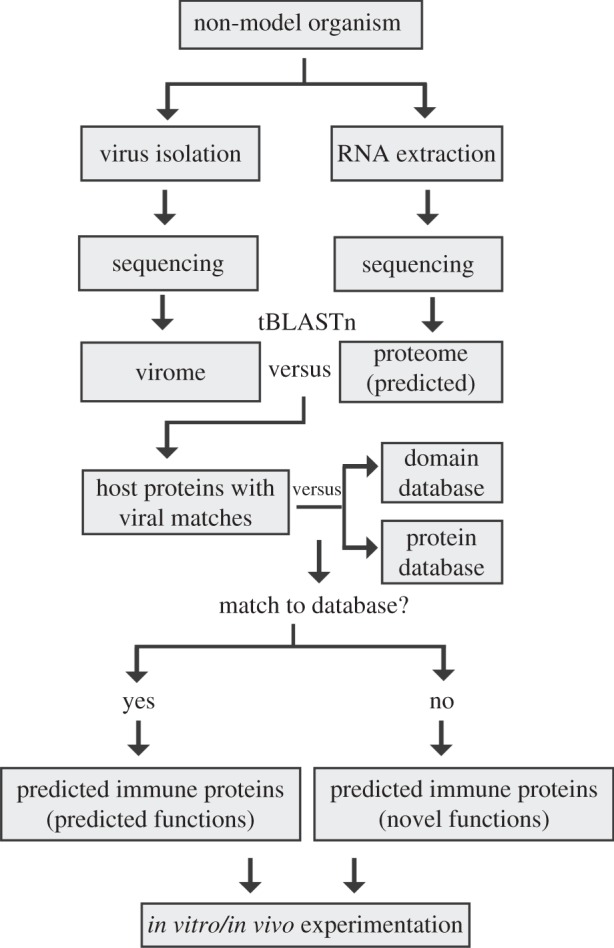
Proposed pipeline to predict immune proteins in uncharacterized systems. Viruses are extracted from the non-model organism of interest using a virus-like particle extraction protocol of choice and nucleic acid is sequenced. If a gene model is not available total RNA is also extracted from host tissue and an assembled transcriptome is prepared. Viral gene segments are compared with the translated transcriptome through tBLASTn to identify matches to host proteins. These proteins are further analysed through comparison with a well-characterized immune system using BLASTp (e.g. human or mouse) and domain prediction database (e.g. conserved domain CDD, Pfam). Proteins that lack hits to either database represent predicted immune genes with unknown function and are selected for further biochemical investigations using *in vitro* and *in vivo* experimentation.

The pipeline described in figure 3 resulted in 159 coral proteins that could not be annotated using either the human proteome or the CDD. To determine the predicted cellular localization (i.e. membrane bound or cytoplasmic) transmembrane regions were predicted using the TMHMM server resulting in 25 proteins possessing 1–14 transmembrane regions (electronic supplementary material, figure S2 and file S5) [31]. Based on the pipeline used in this study a general experimental approach is proposed to identify novel immune components of non-model organisms (figure 5). Viral nucleic acid is isolated from host tissue and compared with the host transcriptome to identify protein candidates that are predicted targets of viral proteins and therefore predicted to be involved with host immunity. This candidate group of immune-associated proteins is then compared with a protein domain database and the proteome of an established model organism to remove proteins whose function can already be predicted. Proteins that lack similarity to either database represent the final group of candidate immune genes with completely unknown function that can be further explored using *in vitro* and *in vivo* experimentation (figure 5).

## 3. Discussion

### (a) Summary

The pipeline described here combines domain-based protein annotations with novel annotations generated by viral communities to predict the host immune repertoire. In animals domain-based methods are biased towards the three major phyla investigated thus far (i.e. Chordata, Nematoda and Arthropoda); therefore, we cannot exclusively rely on those strategies to elucidate the immune systems of uncharacterized phyla [2]. Viral communities provide a domain-independent approach to predict host immune proteins that supplements existing domain-based protein annotation methods.

### (b) Viral manipulation of apoptosis and intracellular pathogen sensing

The domain-based annotation of the coral proteome indicates the coral immune system is highly complex and in some instances more complex than humans [16,32,33]. As expected, many of the components predicted to be involved with coral immunity based on the presence of conserved domains are also predicted targets of the viral community. With the rise of metagenomics, it has become clear that many viruses are persistent and do not cause any known pathologies. For example, the virome of apparently healthy humans includes members of *Herpesviridae*, *Polymovaviridae*, *Papillomavirade*, *Adenoviridae*, *Anelloviridae* and *Parvoviridae* [34–37]. To maintain viral infection over time, persistent viruses will often express multiple proteins that possess sequence similarity to host proteins involved with programmed cell death (apoptosis) and PAMP sensing [9,38]. Figure 4 and electronic supplementary material, figure S1 suggest that, similar to humans, the viral communities associated with apparently healthy coral interact with apoptosis and PAMP sensing. Specifically, the tumour necrosis factor (TNF) signalling pathway and nod-like receptors (NLRs) are predicted targets of coral-associated viruses.

The TNF signalling pathway acts as a central mediator of apoptosis and appears to be functionally conserved from corals to humans [33,39]. TNF-receptor-associated factors (TRAFs) are critical adaptor proteins that bind to the intracellular portion of TNF receptors regulating cell survival and cytokine production [40]. Based on the total number of viral sequences matching to coral proteins homologues of TRAF6, TRAF4 and multiple TNFs were in the top 10% of all host proteins, suggesting that the TNF-signalling pathway is a major target of the coral virome (figure 4; electronic supplementary material, figure S1). In humans, viruses belonging to *Herpesviridae*, *Adenoviridae*, *Reoviridae* and *Retroviridae* have been shown to interact with TNF-mediated apoptosis [8]. Metagenomic evidence in corals indicates that similar to humans, the abundance of herpes viruses changes in response to physiological stress and is associated with bleaching events [41–45]. Future work should focus on which families of viruses interact with the coral TNF-signalling pathway and whether related viral manipulation strategies exist in corals and humans.

NLRs are intracellular signalling molecules that play a central role in the detection of PAMPs [46]. The genome of the reef-building coral *A. digitifera* encodes 500 predicted NLRs compared with only 22 found within the human repertoire [32]. In total, 398 viral hits were distributed across 25 NLRs including many containing a glycosyl transferase domains (electronic supplementary material, figure S1). Corals are the first metazoans described thus far to contain the NLR-glycosyl transferase domain combination; however, the function any coral NLR has yet to be determined. The pipeline described here provides a starting point in the selection of specific NLRs that warrant additional investigation. Taken together, domain-based protein annotation provides a predicted framework of the coral immune system and, based on those annotations, coral-associated viruses are modulating pathogen sensing and the host apoptotic response.

### (c) Beyond conserved domains-novel predictions from viral communities

Domain-based annotation allows for the rapid prediction of host immune proteins. However, the databases used to generate those annotations are largely based on experimental evidence from only three animal phyla (i.e. Chordata, Nematoda and Arthropoda) [2]. While many protein domains are expected to be conserved across phyla, relying exclusively on domain-based annotations will fail to identify immune proteins that lack previously characterized domains. The pipeline described here identified 159 predicted coral immune proteins that could not be annotated using the CDD and lack homology to any human protein. In addition to missing completely novel immune domains, comparing the host proteome to the CDD will also fail to identify proteins involved with immunity that lack canonical immune domains (electronic supplementary material, table S5). In total, 275 coral proteins could be annotated with the CDD but did not contain any PAMP sensing or apoptotic domains or possess any similarity to human proteins (electronic supplementary material, file S5). This group of proteins, as well as the 159 proteins that do not match any domain in the CDD, may be involved in coral-specific immunological processes. The pipeline described here provides a more comprehensive prediction of the host immune repertoire by combining the power of existing databases with new predictions generated from resident viral communities.

### (d) Caveats and future work

One limitation of the proposed bioinformatic pipeline is its reliance upon amino acid alignments long enough to produce a significant *e*-value using tBLASTn. For example, some viruses hijack cell regulation using short elements termed eukaryotic linear motifs (ELMs) that are only two to eight residues in length [47,48]. Therefore, the short alignments between viral ELMs and their target host proteins would probably not produce significant *e*-values and fail to be identified. It is also important to remember that this method generates hypotheses that require direct biological and biochemical validation. This validation would ideally be performed within the organism of interest; however, if molecular techniques remain unavailable then a hybrid approach may be taken to provide a more rapid turnaround of hypothesis testing. For example, the established model organism *Nematostella vectensis* (sea anemone), a fellow anthozoan related to corals, has well-developed molecular methods that could be used to test the function of predicted coral immune proteins [16,49]. While this study has focused on characterizing metazoan immunity, the pipeline proposed here could also elucidate interactions between bacteria and their resident bacteriophage communities to identify novel bacterial immune proteins. Combining these data with metazoan-virus predictions would provide a broad understanding of immune processes occurring within the entire holobiont. The pipeline presented here predicts immune system structure by combining model organism-based databases with a domain-independent approach based on resident viral communities. This method can be applied to any holobiont with the potential to discover novel immunological processes.

## 4. Material and methods

### (a) Isolation of virus-like particles from coral tissue and sequencing of viral DNA

Virus-like particles (VLPs) were isolated from individual colonies of *Po. rus, Acropora* sp. and *P. verrucosa* taken from two locations on the island of Mo'orea, French Polynesia while aquarium samples of *A. yongeii* were generously donated by the Birch Aquarium, San Diego, CA. Sampling locations included the back reef of the Richard Gump Research Station (LT) and Tema'e beach (TA). Briefly, coral tissue was homogenized in the field with a mortar and pestle and 12 ml of 0.02 µm filtered seawater was added until all coral tissue was removed from the skeleton. Next, the coral-tissue slurry was placed in a 15 ml falcon tube followed by the addition of 1 ml of chloroform and stored at 4°C for three weeks. To isolate VLPs from coral tissue, established ultracentrifugation protocols were used [13,50]. Briefly, 600 µl of 50 nmol l^−1^ dithiothreitol was mixed with 8 ml of coral slurry by vortexing, incubated at 37°C for 1 h and centrifuged for 15 min at 3000 r.p.m. In total, 7 ml of the supernatant was then transferred to the top of a CsCl step gradient containing 1.0 ml of each CsCl solution at densities of 1.7 g ml^−1^, 1.5 g ml^−1^ and 1.35 g ml^−1^. The step gradient containing the coral slurry was then spun for 2 h at 22 000 r.p.m. using an SW-41 Ti rotor (Beckman Coulter) and 1.5 ml was extracted from the 1.35–1.5 interface using a 24-gauge needle with an upward-facing tip. Next, the sample was treated with DNase at a final concentration of 100 units ml^−1^ and incubated at 37°C for 1 h. Finally, DNA was extracted from the VLPs using standard CTAB/formamide methods [13]. Two quality control approaches were taken to ensure viral purity of the final DNA product. First, VLPs were visualized before and after ultracentrifugation using standard SYBR-gold staining techniques, followed by a 16S and 18S PCR to ensure the isolated DNA was not contaminated with cellular genetic material [13].

To obtain sufficient nucleic acid for library preparation viral DNA was amplified using a modified linker amplified shotgun library (LASL) method [51]. Briefly, initial DNA concentrations were determined using the Qubit Fluorometric Assay (Life Technologies) and 5 ng of total DNA was combined with PCR-grade water to a final volume of 50 µl. Diluted DNA samples were briefly vortexed and transferred into 50μl Covaris tubes for shearing using the Covaris M220 Focused Ultrasonicator (40 s at 9 W). Sheared DNA was then end-repaired and ligated to LASL Linker A (Linker A FWD: 5′-P-GTATGCTTCGTGATCTGTGTGGGTGT-3′, Linker A REV: 5′-CCACACAGATCACGAAGCATAC-3′) followed by size selection with a target size of 500 base pairs using Pippin Prep (Sage Science) [51]. For each biological sample, four PCR reactions were prepared using a barcoded primer and 17 cycles of PCR were performed (PfuTurbo Cx Hotstart DNA Polymerase, Agilent). Replicates of the same samples were combined, purified and separated into four aliquots for further amplification with three cycles of reconditioning PCR. Finally, all barcoded samples were quantified and pooled for library preparation and sequencing on MisSeq platform using the MiSeq Reagent Kit v3 600 cycles chemistry (Illumina).

### (b) Quality control of sequence data

Preceding downstream analysis quality control of sequence data was performed using a variety of bioinformatic tools. Paired end reads were first joined using COPE [52], low-quality sequences (less than 100 base pairs in length, mean quality score less than 25, or containing more than 10% N's) were removed using PRINSEQ and any remaining bacterial or human sequences were removed with DeconSeq [14,15] (see electronic supplementary material, table S1 for the number of sequences removed at each pre-processing step).

### (c) Bioinformatic pipeline used to analyse viromes

Viromes were compared with the predicted coral proteome [53] using standalone tBLASTn analysis with computational-based statistics turned on (D) and low complexity regions of proteins masked (seq) [17]. Predicted coral proteins were classified as ‘hits’ if five or more viral sequence fragments matched a coral protein with an *e*-value less than 1 × 10^−4^. Predicted coral protein hits were then compared with the human proteome using BLASTp analysis with an *e*-value cutoff of less than 1 × 10^−5^ and the CDD with an *e*-value cutoff of 0.01 [18].

## Supplementary Material

Supplementary Tables

## Supplementary Material

File S1- HumanVirome200bpVSProt.m6_tBLASTn_results

## Supplementary Material

File S2-CoralViromesVSCoralProt.m6_tBLASTn_results

## Supplementary Material

File S3-CoralProtwViralHitsVSHumanProt.m6_BLASTp_results

## Supplementary Material

File S4 - CDD Coral proteins with viral hits no human matches

## Data accessibility

Processed viromes and all supplementary material have been deposited in Dryad: http://dx.doi.org/10.5061/dryad.tb2p4 [54]. All bioinformatics analyses performed are available in electronic supplementary material, file S6 and are available on GitHub under the article title.

## References

[1] Cooper EL 1985 Comparative immunology. Integr. Comp. Biol. 25, 649–664. 10.1093/icb/43.2.278 21680434

[2] Litman GW, Cooper MD 2007 Why study the evolution of immunity? Nat. Immunol. 8, 547–548. (10.1038/ni0607-547) 17514203 PMC3684968

[3] Cooper MD, Peterson RDA, Good RA 1965 Delineation of the thymic and bursal lymphoid systems in the chicken. Nature 205, 143–146. (10.1038/205143a0) 14276257

[4] Frank U, Rabinowitz C, Rinkevich B 1994 *In vitro* establishment of continuous cell cultures and cell lines from ten colonial cnidarians. Mar. Biol. 120, 491–499. (10.1007/BF00680224)

[5] Kopecky EJ, Ostrander GK 1999 Isolation and primary culture of viable multicellular endothelial isolates from hard corals. In Vitro Cell. Dev. Biol. Anim. 35, 616–624. (10.1007/s11626-999-0101-x) 10614872

[6] Helman Y, Natale F, Sherrell RM, Lavigne M, Starovoytov V, Gorbunov MY, Falkowski PG. 2008 Extracellular matrix production and calcium carbonate precipitation by coral cells *in vitro*. Proc. Natl Acad. Sci. USA 105, 54–58. (10.1073/pnas.0710604105) 18162537 PMC2224230

[7] Reyes-Bermudez A, Miller DJ 2009 *In vitro* culture of cells derived from larvae of the staghorn coral *Acropora millepora*. Coral Reefs 28, 859–864. (10.1007/s00338-009-0527-3)

[8] Benedict C 2003 Viruses and the TNF-related cytokines, an evolving battle. Cytokine Growth Factor Rev. 14, 349–357. (10.1016/S1359-6101(03)00030-3) 12787571

[9] Tortorella D, Gewurz BE, Furman MH, Schust DJ, Ploegh HL 2000 Viral subversion of the immune system. Annu. Rev. Immunol. 18, 861–926. (10.1146/annurev.immunol.18.1.861) 10837078

[10] Liang C, Lee J-S, Jung JU 2008 Immune evasion in Kaposi's sarcoma-associated herpes virus associated oncogenesis. Semin. Cancer Biol. 18, 423–436. (10.1016/j.semcancer.2008.09.003) 18948197 PMC7386567

[11] Hagai T, Azia A, Babu MM, Andino R 2014 Use of host-like peptide motifs in viral proteins is a prevalent strategy in host–virus interactions. Cell Rep. 7, 1729–1739. (10.1016/j.celrep.2014.04.052) 24882001 PMC4089993

[12] Alcami A 2003 Viral mimicry of cytokines, chemokines and their receptors. Nat. Rev. Immunol. 3, 36–50. (10.1038/nri980) 12511874

[13] Thurber RV, Haynes M, Breitbart M, Wegley L, Rohwer F 2009 Laboratory procedures to generate viral metagenomes. Nat. Protoc. 4, 470–483. (10.1038/nprot.2009.10) 19300441

[14] Schmieder R, Edwards R 2011 Fast identification and removal of sequence contamination from genomic and metagenomic datasets. PLoS ONE 6, e17288 (10.1371/journal.pone.0017288) 21408061 PMC3052304

[15] Schmieder R, Edwards R 2011 Quality control and preprocessing of metagenomic datasets. Bioinformatics 27, 863–864. (10.1093/bioinformatics/btr026) 21278185 PMC3051327

[16] Shinzato C et al. 2011 Using the *Acropora digitifera* genome to understand coral responses to environmental change. Nature 476, 320–323. (10.1038/nature10249) 21785439

[17] Altschul SF, Gish W, Miller W, Myers EW, Lipman DJ 1990 Basic local alignment search tool. J. Mol. Biol. 215, 403–410. (10.1016/S0022-2836(05)80360-2) 2231712

[18] Marchler-Bauer A et al. 2015 CDD: NCBI's conserved domain database. Nucleic Acids Res. 43(D1), D222–D226. (10.1093/nar/gku1221) 25414356 PMC4383992

[19] Koonin EV, Aravind L 2000 The NACHT family—a new group of predicted NTPases implicated in apoptosis and MHC transcription activation. Trends Biochem. Sci. 25, 223–224. (10.1016/S0968-0004(00)01577-2) 10782090

[20] Strober W, Murray PJ, Kitani A, Watanabe T 2006 Signalling pathways and molecular interactions of NOD1 and NOD2. Nat. Rev. Immunol. 6, 9–20. (10.1038/nri1747) 16493424

[21] Holmskov U, Malhotra R, Sim RB, Jensenius JC 1994 Collectins: collagenous C-type lectins of the innate immune defense system. Immunol. Today 15, 67–74. (10.1016/0167-5699(94)90136-8) 8155265

[22] Medzhitov R 2001 Toll-like receptors and innate immunity. Nat. Rev. Immunol. 1, 135–145. (10.1038/35100529) 11905821

[23] Takahashi M et al. 2008 Mannose-binding lectin (mbl)-associated serine protease (masp)-1 contributes to activation of the lectin complement pathway. J. Immunol. 180, 6132–6138. (10.4049/jimmunol.180.9.6132) 18424734

[24] Aggarwal BB 2003 Signalling pathways of the TNF superfamily: a double-edged sword. Nat. Rev. Immunol. 3, 745–756. (10.1038/nri1184) 12949498

[25] Ashkenazi A, Dixit VM 1998 Death receptors: signaling and modulation. Science 281, 1305–1308. (10.1126/science.281.5381.1305) 9721089

[26] Arch RH, Gedrich RW, Thompson CB 1998 Tumor necrosis factor receptor-associated factors (TRAFs)—a family of adapter proteins that regulates life and death. Genes Dev. 12, 2821–2830. (10.1101/gad.12.18.2821) 9744859

[27] Valmiki MG, Ramos JW 2009 Death effector domain-containing proteins. Cell. Mol. Life Sci. 66, 814–830. (10.1007/s00018-008-8489-0) 18989622 PMC11131443

[28] Weber CH, Vincenz C 2001 The death domain superfamily: a tale of two interfaces? Trends Biochem. Sci. 26, 475–481. (10.1016/S0968-0004(01)01905-3) 11504623

[29] Bouchier-Hayes L, Martin SJ 2002 CARD games in apoptosis and immunity. EMBO Rep. 3, 616–621. (10.1093/embo-reports/kvf139) 12101092 PMC1084193

[30] Lamkanfi M, Festjens N, Declercq W, Vanden Berghe T, Vandenabeele P 2007 Caspases in cell survival, proliferation and differentiation. Cell Death Differ. 14, 44–55. (10.1038/sj.cdd.4402047) 17053807

[31] Krogh A, Larsson B, Von Heijne G, Sonnhammer ELL 2001 Predicting transmembrane protein topology with a hidden Markov model: application to complete genomes. J. Mol. Biol. 305, 567–580. (10.1006/jmbi.2000.4315) 11152613

[32] Hamada M, Shoguchi E, Shinzato C, Kawashima T, Miller DJ, Satoh N 2013 The complex NOD-like receptor repertoire of the coral *Acropora digitifera* includes novel domain combinations. Mol. Biol. Evol. 30, 167–176. (10.1093/molbev/mss213) 22936719

[33] Quistad S, Stotland A, Barott K, Smurthwaite C, Hilton B, Grasis J 2014 Evolution of TNF-induced apoptosis reveals 550 My of functional conservation. Proc. Natl Acad. Sci. USA 111, 9567–9572. (10.1073/pnas.1405912111) 24927546 PMC4084427

[34] Wylie KM, Mihindukulasuriya KA, Zhou Y, Sodergren E, Storch GA, Weinstock GM 2014 Metagenomic analysis of double-stranded DNA viruses in healthy adults. BMC Biol. 12, 2317 (10.1186/s12915-014-0071-7) PMC4177058 25212266

[35] Virgin HW, Wherry EJ, Ahmed R 2009 Redefining chronic viral infection. Cell 138, 30–50. (10.1016/j.cell.2009.06.036) 19596234

[36] Bottalico D et al. 2011 The oral cavity contains abundant known and novel human papillomaviruses from the betapapillomavirus and gammapapillomavirus genera. J. Infect. Dis. 204, 787–792. (10.1093/infdis/jir383) 21844305 PMC3156102

[37] Li J et al. 2012 Nine complete genome sequences of cutaneous human papillomavirus genotypes isolated from healthy skin of individuals living in rural He Nan Province, China. J. Virol. 86, 11936 (10.1128/JVI.01988-12) 23043169 PMC3486313

[38] Hu Z, Usherwood EJ 2014 Immune escape of gamma-herpesviruses from adaptive immunity. Rev. Med. Virol. 24, 365–378. (10.1002/rmv.1791) 24733560 PMC4198523

[39] Micheau O, Tschopp J 2003 Induction of TNF receptor I-mediated apoptosis via two sequential signaling complexes. Cell 114, 181–190. (10.1016/S0092-8674(03)00521-X) 12887920

[40] Häcker H, Tseng P-H, Karin M 2011 Expanding TRAF function: TRAF3 as a tri-faced immune regulator. Nat. Rev. Immunol. 11, 457–468. (10.1038/nri2998) 21660053

[41] Vega Thurber RL et al. 2008 Metagenomic analysis indicates that stressors induce production of herpes-like viruses in the coral *Porites compressa*. Proc. Nat. Acad. Sci. USA 105, 18 413–18 418. (10.1073/pnas.0808985105) PMC2584576 19017800

[42] Marhaver KL, Edwards RA, Rohwer F 2008 Viral communities associated with healthy and bleaching corals. Environ. Microbiol. 10, 2277–2286. (10.1111/j.1462-2920.2008.01652.x) 18479440 PMC2702503

[43] Thurber RLV, Correa AMS 2011 Viruses of reef-building scleractinian corals. J. Exp. Mar. Biol. Ecol. 408, 102–113. (10.1016/j.jembe.2011.07.030)

[44] Wood-Charlson EM, Weynberg KD, Suttle CA, Roux S, Van Oppen MJH In press. Metagenomic characterisation of viral communities in corals: mining biological signal from methodological noise. Environ. Microbiol. Rep. (10.1111/1758-2229.12275) 25708646

[45] Correa AMS, Ainsworth TD, Rosales SM, Thurber AR, Butler CR, Vega Thurber RL 2016 Viral outbreak in corals associated with an *in situ* bleaching event: atypical herpes-like viruses and a new megavirus infecting *Symbiodinium*. Front. Microbiol. 7, 127. 26941712 10.3389/fmicb.2016.00127 PMC4761846

[46] Kersse K, Bertrand MJM, Lamkanfi M, Vandenabeele P 2011 NOD-like receptors and the innate immune system: coping with danger, damage and death. Cytokine Growth Factor Rev. 22, 257–276. (10.1016/j.cytogfr.2011.09.003) 21996492

[47] Davey NE et al. 2012 Attributes of short linear motifs. Mol. Biosyst. 8, 268–281. (10.1039/C1MB05231D) 21909575

[48] Davey NE, Trav G, Gibson TJ 2011 How viruses hijack cell regulation. Trends Biochem. Sci. 36, 159–169. (10.1016/j.tibs.2010.10.002) 21146412

[49] Putnam NH et al. 2007 Sea anemone genome reveals ancestral eumetazoan gene repertoire and genomic organization. Science 317, 86–94. (10.1126/science.1139158) 17615350

[50] Willner D et al. 2009 Metagenomic analysis of respiratory tract DNA viral communities in cystic fibrosis and non-cystic fibrosis individuals. PLoS ONE 4, e7370 (10.1371/journal.pone.0007370) 19816605 PMC2756586

[51] Duhaime MB, Deng L, Poulos BT, Sullivan MB 2012 Towards quantitative metagenomics of wild viruses and other ultra-low concentration DNA samples: a rigorous assessment and optimization of the linker amplification method. Environ. Microbiol. 14, 2526–2537. (10.1111/j.1462-2920.2012.02791.x) 22713159 PMC3466414

[52] Liu B et al. 2012 COPE: An accurate k-mer-based pair-end reads connection tool to facilitate genome assembly. Bioinformatics 28, 2870–2874. (10.1093/bioinformatics/bts563) 23044551

[53] Koyanagi R, Takeuchi T, Hisata K, Gyoja F, Shoguchi E, Satoh N, Kawashima T. 2013 MarinegenomicsDB: an integrated genome viewer for community-based annotation of genomes. Zool. Sci. 30, 797–800. (10.2108/zsj.30.797) 24125644

[54] Quistad SD, Lim YW, Silva GGZ, Nelson CE, Haas AF, Kelly LW, Edwards RA, Rohwer FL 2016 Data from: Using viromes to predict novel immune proteins in non-model organisms. Dryad Digital Repository. (10.5061/dryad.tb2p4) PMC5013795 27581878

